# Conflict Identification and Zoning Optimization of “Production-Living-Ecological” Space

**DOI:** 10.3390/ijerph19137990

**Published:** 2022-06-29

**Authors:** Pengnan Xiao, Jie Xu, Chong Zhao

**Affiliations:** 1 College of Urban and Environmental Sciences, Central China Normal University, Wuhan 430079, China; maikedang@mails.ccnu.edu.cn; 2 Faculty of Resources and Environmental Science, Hubei University, Wuhan 430062, China; 201901110800086@stu.hubu.edu.cn; 3 School of Chemistry and Environmental Engineering, Wuhan Polytechnic University, Wuhan 430040, China

**Keywords:** “production-living-ecological” space, space conflict, FLUS model, land use change

## Abstract

With the acceleration of economic and social development and the increasing competition between multi-functional spaces, the coordination and stability of land space have been seriously affected. In order to simulate the conflict pattern of “production, living ecological” space and analyze its evolution characteristics, taking Qianjiang City as the research area and based on the current data of land use, the FLUS (Future Land Use Simulation) model and spatial conflict measurement model are used to calculate the change trend of “production, living ecological” spatial conflict in Qianjiang City in the past and in the future. The research results are of great significance for the scientific use of land space and the optimization of regional development patterns. The results show that: (1) From 2000 to 2020, the level of spatial conflict in Qianjiang City showed an upward trend, the proportion of medium and above conflict units gradually increased, and the conflict level in the study area gradually became dominated by strong conflict. (2) Due to the process of urbanization and the continuous growth of population and GDP (Gross Domestic Product), the construction land in Qianjiang City shows a rapid increase trend under three scenarios, and the cultivated land area shows a downward trend. (3) In 2035, under the three scenarios, the spatial conflict in Qianjiang City will be strengthened, mainly at the level of medium and above. (4) According to the change degree of conflict transformation, 15 change types are divided into five functional zones: ecological protection zone, ecological conservation zone, modern agriculture zone, urban–rural development coordination zone and urban optimization zone.

## 1. Introduction

The gradual acceleration of industrialization and urbanization has led to an unprecedented impact to the layout of land spatial patterns, and makes the development of land spatial patterns face severe challenges and crises, such as the annual growth of urban and rural construction land, the extrusion of agricultural and ecological space, serious environmental pollution, the degradation of ecosystem, and the intensification of the contradiction between urban, agricultural and ecological space [1]. Improving the distribution pattern of land and space, realizing the sustainable development of ecological space, intensive production space and suitable living space, and realizing the concept of integrated development of land and space have attracted extensive attention from national government departments and academia [2]. As the most direct and important goal and expression of land space development and governance under the concept of ecological civilization construction, and an important starting point for realizing the optimization of land space development patterns, the research on the classification and adjustment of “production-living-ecological” spatial structure and its constituent elements has become an urgent practical problem to be solved in the current academic frontier and land space planning [3].

To date, the researchers have conducted a lot of research on the spatial identification of “production-living-ecological” and made a series of achievements, mainly focusing on the concept and connotation of “production-living-ecological” space [1,3,4,5], land classification systems of “production-living-ecological” space [6,7,8,9],” spatial identification of “production-living-ecological” space [10,11,12,13] and “production-living-ecological” space optimization [14,15,16,17]. The concept and theory of “production-living-ecological” space are widely used in various fields of land science, including “production-living-ecological” space and ecosystem service value [18,19], human settlements [20,21,22], land remediation [23,24,25], land space planning [26,27,28], etc. In recent years, the diagnosis and problem analysis of “production-living-ecological” space conflict is not only the direction scholars are concerned by, but also the key link and important step to identify the intensity of land space conflict and existing problems. The fundamental purpose of analyzing spatial relations, such as spatial competition, conflict and disharmony, is to provide solutions to the problems caused by disorderly development, over development and decentralized development, including excessive occupation of high-quality cultivated land, ecological damage and environmental pollution [1]. Spatial conflict is due to the scarcity of land resources and the spillover of functions, and the multi suitability of land resource utilization, the fixity of spatial location and the overlap and competition of benefits of various stakeholders are the causes of spatial conflict [29]. The research on conflict intensity measurement methods contains important research content of conflict diagnosis [30,31]. There are two mainstream measurement methods. The first is the mathematical statistical analysis method, including the “pressure-state-response” model [32], and the “risk-sustainability-vulnerability-resilience of the insured body” [33] from the perspective of economics. The second is the spatial analysis method, which is a method to measure the conflict intensity by constructing the conflict index (comprehensive index and type index) with the help of landscape patch characteristic parameters [34,35], or a method to identify and quantitatively measure the type and intensity of land use conflict by using the arrangement and combination method of different land suitability levels and competitiveness intensity levels [35]. These methods are widely applied in the study of land use conflict measurement and provide a large number of cases and rich experience for the diagnosis and problem analysis of “production-living-ecological” spatial conflict. Although scholars continue to deepen the exploration of land use conflict, some deficiencies still exist. In terms of research scale, there are many studies on urban agglomeration [2,36] and prefecture level cities [37], but there are few studies on small and medium-sized scales, such as counties and towns. In terms of research content, the analysis of space use conflict is less [38], and the existing space use conflict is mostly concentrated in landscape space, and the exploration of “production-living-ecological” space use conflict is rarely seen. In terms of time scale selection, it mainly focuses on current situation research, and there is little research on predicting the change of conflict pattern [39].

The innovation of this paper is that on the basis of dividing the “production-living-ecological” space and taking the grid as the evaluation unit, the spatial conflict model and the FLUS (Future Land Use Simulation) model are comprehensively used to analyze the past, present and future land use conflicts in Qianjiang City, so as to realize the early warning of spatial conflicts.

This paper can provide support and basis for Qianjiang City to alleviate the “production-living-ecological” spatial conflict and formulate the optimal allocation policy of land space and provide reference for the coordination and management of the relationship between socio-economic development and ecological environment protection.

## 2. Materials and Methods

### 2.1. Research Area

**Figure 1:**
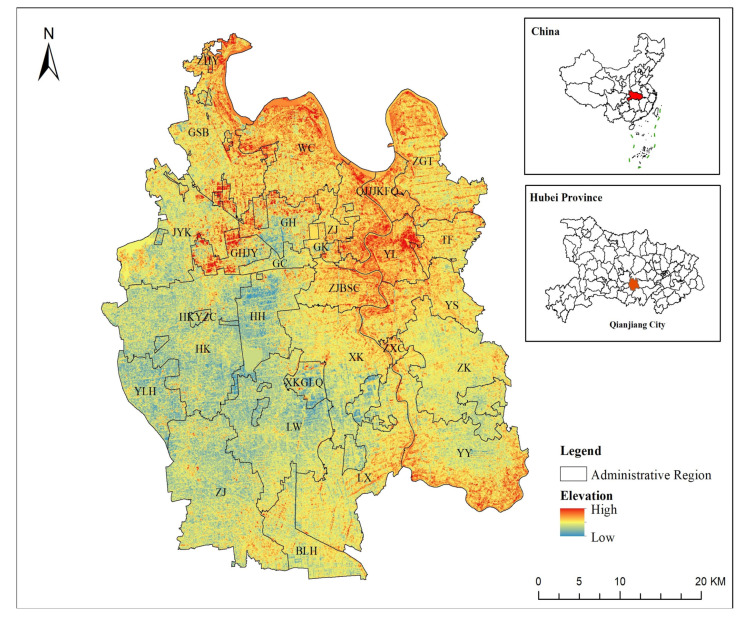
The administrative division and location of research area.

Qianjiang City situated in the central and southern part of Hubei Province, China (Figure 1). It is connected with Hanjiang River in the north, the Yangtze River in the south, Wuhan in the east, Jianli in the south, Jingzhou in the west, Jingmen in the north, facing Tianmen across the Hanjiang River. Shanghai Chengdu Expressway and national highway 318 cross the whole territory, and Shanghai Wuhan Chengdu high-speed railway (HanYi high-speed railway) crosses the east and west. The city governs 15 towns, markets and offices, 1 provincial economic and technological development zone and 6 management zones.

The terrain in Qianjiang City is flat. The terrain is high in the northeast and low in the southwest. The highest point in the territory is 39.77 m, and the lowest point is 28.75 m. The natural gradient of the ground is 1/4000. Qianjiang River is a plain area, and its sediments are mainly modern river alluvium, with a relatively single geomorphic type. The landform roughly includes six types: beach flat, middle flat, low humidity flat, high elevation flat, flat hill and silt flat.

### 2.2. Research Method

#### 2.2.1. Classification System of “Production-Living-Ecological” Space

This paper divides the land use type into “living-production” space, “production-ecological” space, “ecological-production” space, and ecological space (Table 1). Among them, “living-production” space refers to the land space that can provide living functions or production functions other than agricultural production [9], including construction land used for living, urban and village residential land, industrial and mining land, etc. This kind of land is mainly composed of buildings and structures, and there is no clear space boundary, so it can be regarded as “living-production” space. The “production-ecological” space refers to the land space, including cultivated land and garden land, that mainly focuses on agricultural production and can provide ecological functions [3]. In addition to providing agricultural products, these two types of land can also provide ecological functions, such as soil erosion control and climate regulation, so they are classified as “production-ecological” space. The “ecological-production” space refers to the land space that not only has important ecological functions, but also can engage in certain agricultural production, including forest land and water area. This kind of land space not only can help prevent wind and sand, conserve water sources, and purify the environment [6], but also has certain production functions, including providing raw wood materials and aquatic products. Therefore, forest land and water areas can be classified as “ecological-production” spaces. Ecological space refers to the space with the main function of providing ecological products or ecological services. Referring to the spatial division results of unused land by many scholars [7,8], combined with the fact that other grasslands are the main types of unused land in Qianjiang City, the unused land is classified as ecological space.

#### 2.2.2. Spatial Conflict Measurement Model

The essence of spatial conflict is an objective geographical phenomenon, produced by the competition of space resources in the process of a “man-land” relationship, which is not only the consequence of the interaction between natural factors and human factors, but also the key factor influencing regional sustainable development [2]. In terms of the spatial conflict measurement index, based on the ecological theory, some scholars construct the landscape ecological index from the perspective of spatial complexity, spatial vulnerability, and spatial stability [37]. This method expresses the transformation of spatial types and the changes of landscape environment caused by the results of spatial conflict, and the data are easier to obtain. Other scholars, based on the theory of geography, build a spatial conflict measurement model based on the spatial type, spatial pattern, and spatial process [40]. This method is helpful to analyze the problems of unbalanced proportions of spatial structures and improper combination of types, which makes it difficult to obtain data. Other scholars calculate the risk of conflict and analyze the causes of spatial conflict from the economics perspective [33]. Therefore, this paper uses the landscape ecological index based on ecological theory to evaluate the conflict intensity of regional “production-living-ecological” space utilization [30,41]. The spatial conflict composite index (*SCCI*) is expressed by spatial complexity index (*SCI*), spatial vulnerability index (*SVI*), and spatial risk index (*SRI*) [39]:

*SCCI* = *SCI* + *SVI* − *SRI* (1)

The degree of spatial complexity is expressed by spatial complexity index (*SCI*). In the continuous development of economy and society, the increase in land use intensity and the complexity of patch shape lead to the intensification of spatial use contradiction. Therefore, the Area Weighted Mean Patch Fractal Dimension (*AWMPFD*) is applied to quantify the space external pressure. The index shows the impact of neighborhood patches on the measured patches, which directly reflects the interference of human activities on the spatial pattern. A high value indicates that the plaque is under high external pressure. The calculation formula is as follows:

(2) $$AWMPFD=\sum_{i=1}^{m}\sum_{j=1}^{n}\frac{2\ln\left(0.25P_{ij}\right)}{\ln a_{ij}}\times \frac{a_{ij}}{A}$$

where $P_{ij}$ represents the perimeter of the *j*-th patch of the *i*-th spatial type, $A_{ij}$ represents the *j*-th patch area of the *i*-th spatial type and *A* represents the area of the space unit.

Spatial vulnerability index (*SVI*) reflects the capability of measured patches to counter external pressure. If the resistance is weak, it indicates that it is vulnerable to external influence, resulting in a higher level of space conflict. The “production-living-ecological” space is the re-division of landscape land space, and the strength of its internal landscape compression capacity directly affects the spatial vulnerability. Therefore, the spatial vulnerability index is measured by the vulnerability of various landscapes in the space. With reference to relevant literature, the vulnerability of each landscape type is assigned: “living-production” space-4, “production-ecological” space-3, “ecological-production” space-2, and ecological space-1. The calculation formula is as follows:

(3) $$SVI=\overset{m}{\underset{i=1}{\sum }}\overset{r}{\underset{s=1}{\sum }}f_{is}\times \frac{a_{is}}{A}\;$$

where $F_{is}$ represents the perimeter of the *s*-th patch of the *i*-th space type, $a_{is}$ represents the landscape patch area of the *s*-th landscape land type of the *i*-th space type, a represents the space unit area, *m* represents the number of space types, *n* represents the number of patches and *r* represents the landscape land type.

Spatial stability is measured by spatial risk index (*SRI*). If the space form is more fragmented, the space risk will be greater and the stability will be worse, resulting in the higher intensity of space conflict. Therefore, the degree of spatial risk is measured by landscape fragmentation index. The calculation formula is as follows:

(4) $$SRI=1-\frac{PD-PD_{min}}{PD_{max}-PD_{min}}\;$$

where *PD* refers to landscape fragmentation, and $PD_{min}$ and $PD_{max}$ are the minimum and maximum values of landscape fragmentation, respectively. *PD* = *n*/*A*, *A* represents the area of space unit, and *n* represents the number of space types.

This paper takes grid as the evaluation unit. After considering the research scale, the scope of the research area, the volume of data, the status of spatial patches and comparing the grid units of different sizes (including 800 m × 800 m, 900 m × 900 m, 1000 m × 1000 m, 1200 m × 1200 m), it is found that the grid of 1000 m × 1000 m can better reflect the distribution characteristics of spatial conflict, and the data processing capacity is more appropriate. Therefore, 2193 spatial units are divided. Among them, if the patches in the boundary area of the study area are not covered with the whole unit area, they are calculated according to a complete unit area, so as to calculate the above indexes in each spatial unit to quantitatively evaluate the level of spatial conflict. In this study, the spatial conflict index is standardized to 0–1, and the conflict level is divided into five levels by equal spacing method, namely weak spatial conflict [0.0, 0.2], weak spatial conflict [0.2, 0.4], medium spatial conflict [0.4, 0.6], strong spatial conflict [0.6, 0.8] and strong spatial conflict [0.8, 1.0].

#### 2.2.3. Land Use Change Simulation Model

GeoSOS-FLUS model, developed by the Li, is not only applicable to the scenario simulation of land use change in the future [42], but also an effective model for urban development boundary identification and spatial optimization [42]. The model first uses an Artificial Neural Network (ANN) to obtain the suitability probability of various land use types, and then improves the applicability of the model by coupling a System Dynamics model (SD) and Cellular Automata (CA) model. An adaptive inertial competition mechanism in the CA model is introduced to handle the complexity and uncertainty of mutual transformation of various land use types under the joint influence of natural and human factors [42].

(1) Prediction of land demand scale. Under the condition of multi scenario simulation, the future demand of ecological space is inconsistent, and there are also differences in the demand scale of various land types within the “production-living-ecological” space. Therefore, before simulating the future distribution of ecological space, it is necessary to predict the demand scale of each land type according to different scenarios. In this paper, the Markov model is applied to predict the demand scale of various land types in the study area in the future. In the study of land use change, a Markov model realizes the simulation of land use change by assuming that the state of a land use type at *t + 1* is only related to the state of land use type at *t*. The specific process is as follows:

(5) $$S_{\left(t+1\right)}=P_{ab}\times S_{\left(t\right)}$$

where when $S_{\left(t\right)}$ and $S_{\left(t+1\right)}$ are *t* and *t* + 1, the state matrix of land use type in the study area; $P_{ab}$ represents the transition probability matrix from type *a* to type *b*.

(2) Calculation of suitability probability based on ANN. Artificial Neural Network algorithm (ANN) is composed of input layer, hidden layer, and output layer, which includes prediction and training stage. The calculation formula is:

(6) $$\begin{array}{c} sp\left(p,k,t\right)=\sum_{j}\omega_{j,k}\times sigmoid\left(net_{j}\left(p,t\right)\right)\\ =\sum_{j}\omega_{j,k}\times \frac{1}{1+e^{-net_{j}\left(p,t\right)}} \end{array}$$

where $sp\left(p,k,t\right)$ is the suitability probability of *k*-type land under time *t* and grid *p*, $\omega_{j,k}$ is the weight between the output layer and the hidden layer, sigmoid() is the excitation function from the hidden layer to the output layer, $net_{j}\left(p,t\right)$ represents the signal received by the *j*-th hidden layer grid *P* at time *t*. The suitability probability of each land type output by neural network algorithm is 1:

(7) $$\underset{k}{\sum }sq\left(p,k,t\right)=1$$

(3) The mechanism of adaptive inertial competition. The land use transformation probability not only depends on the distribution probability of the output of the neural network, but also is influenced by other factors, including neighborhood density, inertia coefficient, transformation cost, and land type competition. The gap between the current land quantity and land demand will be adjusted adaptively in the iterative process, which determines the inertia coefficient of different land types. The adaptive inertia coefficient ${\;\mathrm{Intertia}\;}_{k}^{t}$ of the *k*-th land type at time *t* is:

(8) $${\mathrm{Intertia}}_{k}^{t}\left\{\begin{array}{l} {\;\mathrm{Intertia}\;}_{k}^{t-1}\;\left|D_{k}^{t-2}\right|⩽\left|D_{k}^{t-1}\right|\\ {\;\mathrm{Intertia}\;}_{k}^{t-1}\times \frac{D_{k}^{t-2}}{D_{k}^{t-1}}\;\;0>D_{k}^{t-2}>D_{k}^{t-1}\\ {\;\mathrm{Intertia}\;}_{k}^{t-1}\times \frac{D_{k}^{t-1}}{D_{k}^{t-2}}\;\;D_{k}^{t-1}>D_{k}^{t-2}>0 \end{array}\right.$$

where $D_{k}^{t-1}$ and $D_{k}^{t-2}$ are the difference between the demand quantity at t−1 and t−2 and the grid quantity in the *k*-th type of land use, respectively.

The CA model is applied to determine each land type iteratively after calculating the probabilities of different grids. At time *t*, the probability of converting grid *p* into *k* land type can be expressed as:

(9) $${\;\mathrm{TProb}\;}_{p,k}^{t}=sp\left(p,k,t\right)\times \Omega_{p,t}^{t}\times {\;\mathrm{Intertia}\;}_{k}^{t}\times \left(1-sc_{c\to k}\right)$$

where $sc_{c\to k}$ is the cost of changing the land type from *c* to *k*, $1-sc_{c\to k}$ is the difficulty degree of conversion, $\Omega_{p,t}^{t}$ is neighborhood action, and its formula is:

(10) $$\Omega_{p,t}^{t}=\frac{\sum_{N\times N}\mathrm{con}\left(c_{p}^{t-1}=k\right)}{N\times N-1}\times Z\omega_{k}$$

where $\sum_{N\times N}\mathrm{con}\left(c_{p}^{t-1}=k\right)$ indicates in *N* × *N*’s Moore neighborhood window, the total number of grids of the *k*-th land class after the last iteration. $\omega_{k}$ is the neighborhood action weight of various land use type.

### 2.3. Data Source and Technical Process

**Figure 2:**
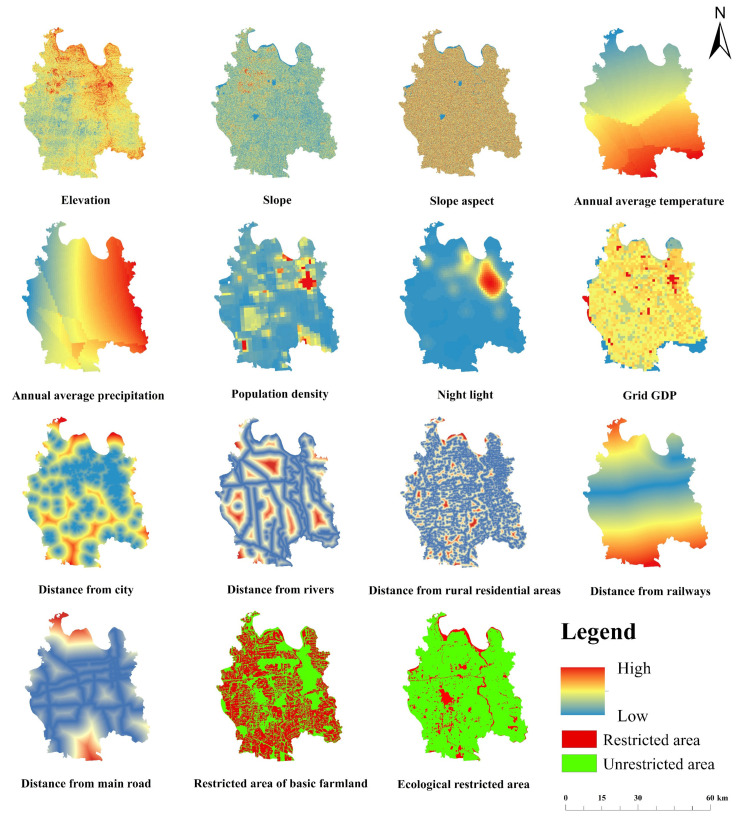
Influencing factor for simulating land use type change.

**Figure 3:**
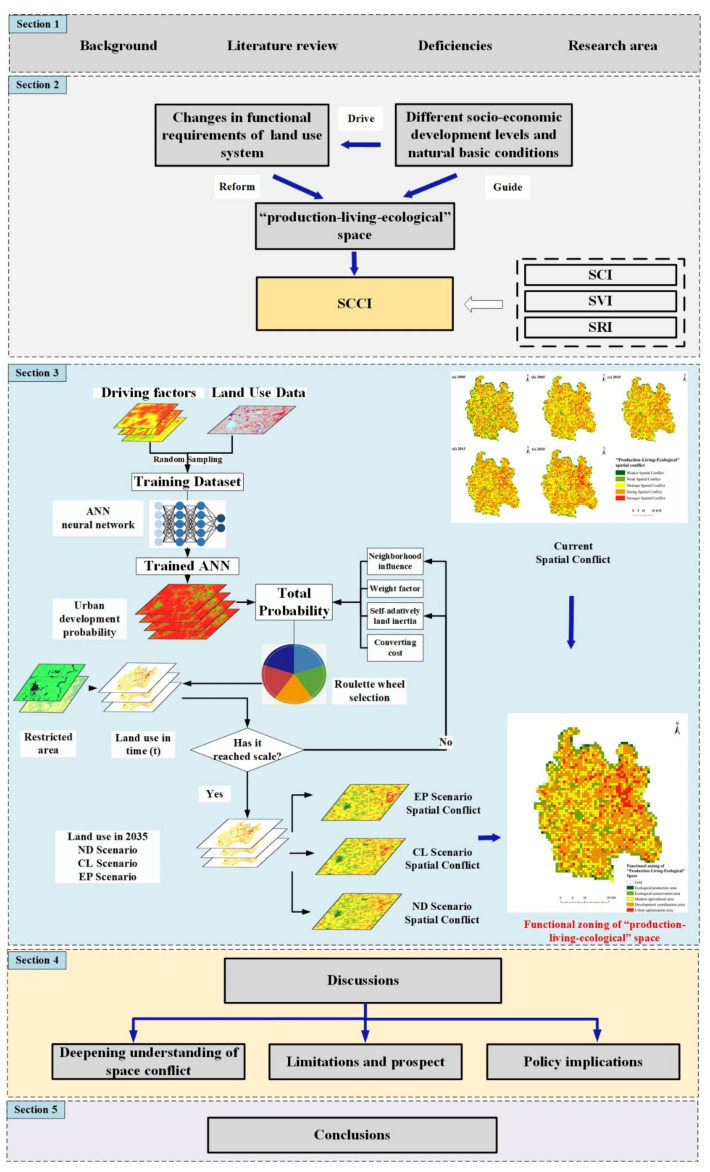
Technical flowchart of research.

The land use data are provided by the Resources and Environmental Sciences Data Center, founded by the Chinese Academy of Sciences (RESDC). The land use types in this set of data include 6 primary types, namely cultivated land, forest land, grassland, water area, construction land, and unused land, according to land resources and their use attributes. Secondary types are mainly divided into 25 types. Therefore, the process of land use remote sensing interpretation includes the formulation and selection of land use/cover remote sensing monitoring and classification system, the selection and processing of remote sensing data sources, the processing of auxiliary data sources, such as topographic maps, the formulation of remote sensing interpretation marks and interpretation principles, the quality inspection and accuracy analysis of interpretation results, etc. According to the verification results, 18 land use/cover types in 421 map spots are wrong, and the remote sensing interpretation accuracy of land use/cover types is 95.72%. DEM data are obtained from a geospatial data cloud platform. The socio-economic data come from the WorldPoP database, National Meteorological Science Data Center, and Land Use Planning Database (2006–2020). Table 2 shows the specific sources of data. Figure 2 shows the influencing factors of simulated land use type change. Figure 3 is the technical flow of the whole article.

## 3. Results

### 3.1. Land Use Change Analysis

**Figure 4:**
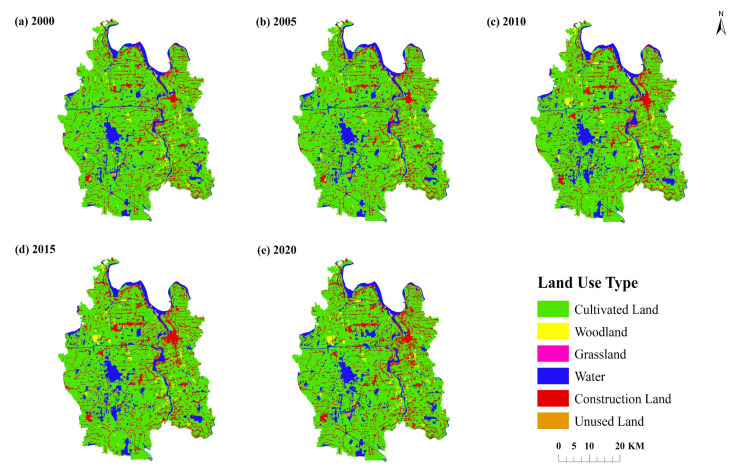
Land use types in Qianjiang City (2000–2020).

Based on the land use data of Qianjiang City (see Figure 4 and Table 3), the cultivated land area of Qianjiang City is vast and widely distributed. The forest land is scattered, mainly around the river shoreline and other water areas. The grassland area is the smallest, showing the characteristics of point distribution in Qianjiang City. In addition to the Han River passing through, there is also a large number of large and small lakes and water systems scattered in the water area. Construction land is mainly concentrated in the northeast of Qianjiang City. The growth of construction land reflects the accelerated urbanization trend of Qianjiang City. Unused land is mainly distributed near the waters in the south.

From the change quantity, the cultivated land area of Qianjiang City decreased the most from 2000 to 2020, with a decrease of 64.21 km^2^. Forest land increased by 2156 km^2^ and grassland decreased by 0.05 km^2^. The water area increased by 21.89 km^2^. The area of unused land changed little. The area of construction land increased the most, with an increase of 35.32 km^2^. In terms of change rate, from 2015 to 2020, grassland changed the most, with a growth rate of 78.87%. Meanwhile, the change range of cultivated land, forest land, grassland and water area were also large, with the change rates of 4.11%, 25.50% and 11.39%, respectively. From the perspective of phased changes, the cultivated land and grassland area show the characteristics of different scales and continuous reduction. The water area and construction land area continue to grow steadily, and the unused land shows a wave type of “decrease-increase-decrease”.

### 3.2. Analysis of Spatiotemporal Changes of “Production-Living-Ecological” Space

#### 3.2.1. Spatial Pattern of “Production-Living-Ecological” Space

**Figure 5:**
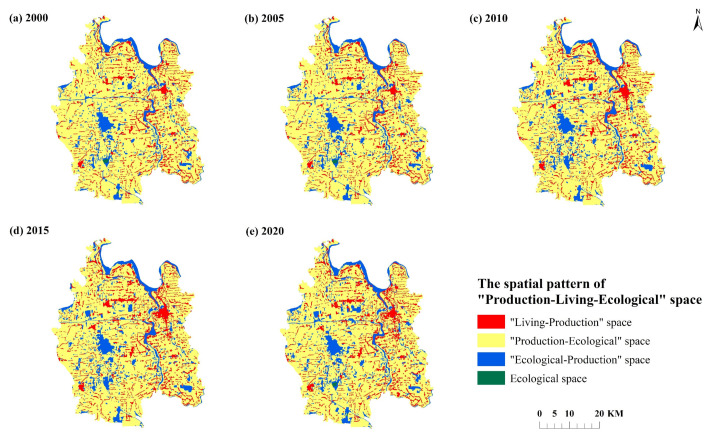
Spatial distribution of “production-living-ecological” space in Qianjiang City.

According to the spatial distribution of “production-living-ecological” space (Figure 5), the annual average of 2005, 2015, and 2020 is dominated by “production-ecological” space, which is distributed in a centralized and continuous manner. The “living-production” spaces are distributed in clusters, and an obvious agglomeration is formed with Qianjiang City as the center. From 2000 to 2020, with the acceleration of urbanization in Qianjiang City, the “living-production” space gradually expanded to the surrounding regional space. The “ecological-production” space is mainly distributed in the northeast of the county and the county boundary zone in the west and south. Due to the occupation of the “production-ecological” space, the spatial pattern shows a shrinking trend. The ecological space is scattered, and the patch area is small. In particular, from 2005 to 2015, the ecological space was largely occupied by “production-ecological” space and “living-production” space, and the space area decreased significantly.

**Figure 6:**
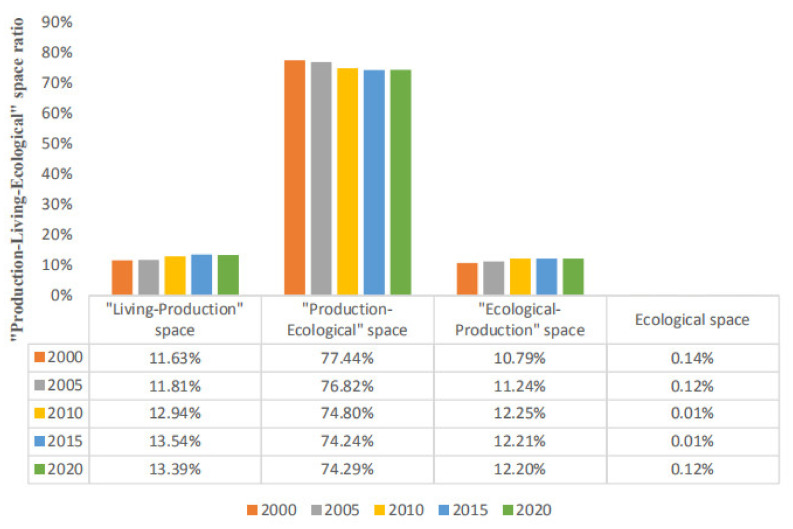
Quantitative change of “production-living-ecological” space in Qianjiang City.

In terms of quantity structure (Figure 6, Table 4), the proportion of “production-ecological” space is the highest from 2000 to 2020, exceeding 74% of the total land area of Qianjiang City. The second is “living-production” space and “ecological-production” space, with their total area accounting for 22–24%. The area of ecological space is the smallest, less than 0.15% of the total area. Moreover, the four types of space show the characteristics of “two rises, one falls and one fluctuation”. The area of “living-production” space shows an upward trend, and the proportion of space area increased from 11.63% in 2000 to 13.39% in 2020. The area of “ecological-production” space and ecological space showed a downward and upward trend, respectively. The area proportion decreased from 77.44 in 2000 to 74.29% in 2020 and increased from 10.79% to 12.20%. The ecological space area shows the characteristics of fluctuation, which shows the trend of decreasing first and then increasing.

#### 3.2.2. Analysis on the Change of “Production-Living-Ecological” Space Conflict

**Figure 7:**
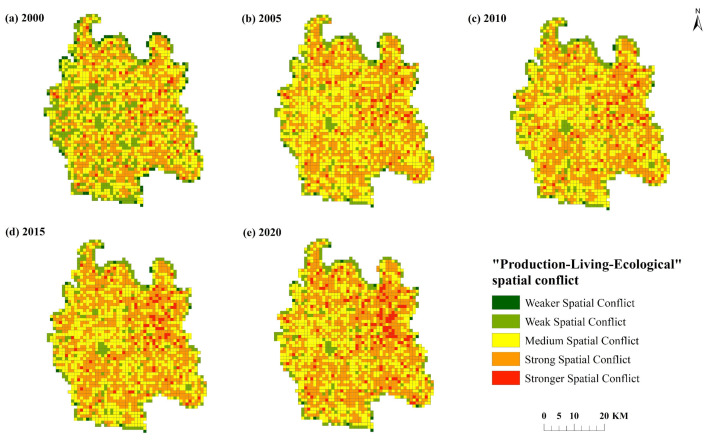
Changes of spatial conflict types in Qianjiang City.

The comprehensive index of “production-living-ecological” spatial conflict in Qianjiang City in 2000–2020 (see Table 5 and Figure 7) is calculated by using the “production-living-ecological” spatial conflict measurement model. The results show that from 2000 to 2010, the spatial conflict in Qianjiang City is mainly at the level of medium and below, and the spatial conflict unit accounts for 54.91%. Among them, the weak spatial conflict is mainly distributed at the county boundary, and the weak spatial conflict units are evenly distributed in blocks, mainly due to the relatively single land type in the spatial unit, especially the weak spatial conflict. The land type in the grid unit is mainly cultivated land, the vulnerability of landscape land type is small, the spatial vulnerability index is low, and the level of spatial conflict is relatively low.

Medium spatial conflict units are distributed in all villages and towns, which are mainly affected by the stability and vulnerability of spatial patches, and the level of spatial conflict is relatively high. The conflict area is concentrated in the eastern part of the town center and the southern part of the city center. Combined with the spatial distribution map of “production-living-ecological” space, these two types of spatial conflict units mainly appear near the county’s “living-production” spaces, which are greatly affected by the development of urbanization. The fragmentation and shape complexity of spatial patches are more prominent than other spatial units, and the intensity of spatial conflict is higher. From 2015 to 2020, the level of spatial conflict in the county increased significantly, dominated by strong spatial conflict, and the number of spatial units accounted for 42.23%. This conflict pattern is mainly affected by the continuous outward expansion of construction land, resulting in the increase in spatial complexity and the level of spatial conflict. The number of strong spatial conflict units has increased significantly, and the concentration intensity is relatively high in the center of Qianjiang City and near all villages and towns.

Overall, from 2000 to 2020, the spatial conflict level in Qianjiang City showed an upward trend, the unit proportion of medium and above conflicts gradually increased, and the conflict level in the study area was gradually dominated by strong conflicts. Strong spatial conflict units gather significantly in the center of Qianjiang City and are concentrated near the “living-production” space. As time goes on, the degree of agglomeration continues to improve. The changes of these two types of spatial conflict patterns are mainly affected by the rapid development of urbanization and industrialization, the continuous expansion of “production-ecological” space and “living-production” space, and the improvement of the fragmentation and complexity of spatial patches, resulting in the improvement of the level of spatial conflict. Qianjiang City is located in the hinterland of Jianghan Plain. The integrated development of Wuhan urban agglomeration will bring greater opportunities for the industrial development of Qianjiang City. The transformation of “living-production” space and “production-ecological” space will be more active, and the “ecological-production” space and ecological space will face threats.

Table 6 reflects the regional characteristics of spatial conflict distribution. Weak spatial conflict mainly occurs in the center of “living-production” space and “production-ecological” space. Due to the low vulnerability of “living-production” space and “production-ecological” space, the complexity of regional space in the center is low and the external pressure is small, so the value of spatial conflict comprehensive index is the lowest. The weak spatial conflict areas are mainly distributed in the boundary areas of different land types with little difference in vulnerability and low spatial complexity. The “production-ecological” space with a large patch area is located in the area with medium spatial conflict levels, due to its high vulnerability and low spatial complexity. Strong spatial conflicts are mostly distributed in areas with complex landscape spatial structures, high spatial vulnerability and a high degree of fragmentation, mainly in “living-production” space and “production-ecological” space. Strong spatial conflicts are mainly distributed in “living-production” space, “production-ecological” space and “ecological-production” space.

**Figure 8:**
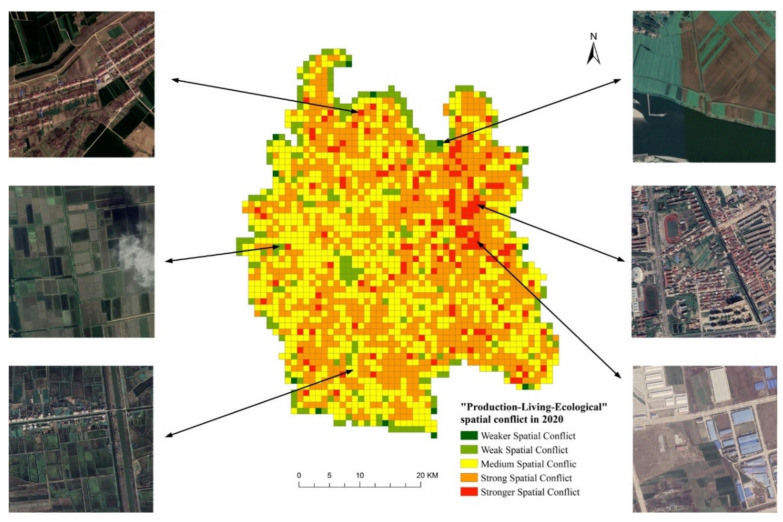
Comparison of spatial conflict level and remote sensing images of “production-living-ecological” in Qianjiang City in 2020.

According to the measurement results of spatial conflict levels in Qianjiang City in 2020 and the regional distribution of each controllable level of conflict, with the help of Google Earth’s remote sensing image platform, select several typical areas with serious out of control and basically out of control conflict levels for remote sensing image screenshot comparison (the captured remote sensing image was taken in December 2020). It was found that the remote sensing images of areas with strong spatial conflict and strong spatial conflict basically reflect some spatial conflict phenomena to varying degrees (as shown in Figure 8). In the calculation results of spatial conflict, the remote sensing images of Qianjiang City Center and Qianjiang Industrial Park, which are in strong spatial conflict and strong spatial conflict, show obvious characteristics of urban built-up areas and urban–rural transition areas. The images show that a large area is in the developed and under development and construction, and the spatial competition between urban and rural space, construction land and agricultural land, is fierce. A large number of ecological spaces are swallowed up by construction land space. In the spatial conflict measurement results, they all belong to the medium conflict level, and their images show that the types of land, such as cultivated land and water, are seriously divided by rural residential areas and urban road network. The land use type in the area with weak spatial conflict is relatively single, which is basically continuous cultivated land. The weak space conflict area contains ecological land, such as rivers and other water bodies.

### 3.3. Simulation of Spatial Conflict of “Production-Living-Ecological” Space in Multi-Scenario

#### 3.3.1. Model Reliability Test

Based on the spatial data of land use in Qianjiang City in 2000, the parameters, such as impact factors, neighborhood weights and conversion rules, are input into the flux model to obtain the land use simulation results in 2005. Based on the current data of land use in 2005, the precision validation module is used to test, and the Kappa coefficient is 0.9114, which shows that the accuracy and practicability of the model are good. The FLUS model can be used to simulate and predict the spatial distribution of land use in Qianjiang City in the future.

#### 3.3.2. Multi-Scenario Scheme Setting

According to the regional situation, resource characteristics, development strategy and future land demand of Qianjiang City fully consider the impact of natural environment, socio-economic and policy factors on future land use change, and set up natural development, cultivated land protection and three land use scenarios of ecological protection. According to the land use structure and pattern under different policy scenarios, the future scenario simulation of land use in 2035 is carried out, and the response characteristics of natural environment and human activities to land use change under different scenarios are analyzed (Table 7), which plays an important role in formulating the corresponding coordinated development strategy of ecological protection and economic construction.

Scenario 1 (Natural development scenario): under the background of rapid urbanization, give full play to the land use benefits with high economic output potential, take economic benefits as the main development goal, accelerate the integrated development of urban and rural areas, promote the steady increase in urbanization rates, increase urban infrastructure construction, increase the land area for transportation and water conservancy construction, and realize the rapid development of regional economy in Qianjiang City. Under the natural development scenario, Qianjiang City mainly takes economic benefits as the priority development goal. Therefore, all land use types are set to be convertible into construction land. The cost of converting construction land to other land use types is high and the possibility is low. The construction land is set as non-convertible to other land types, while the other land use types are converted to each other according to the actual situation.

Scenario 2 (Cultivated land protection scenario): based on the overall land use plan of Qianjiang City (2006–2020) and the basic farmland protection plan of Qianjiang City, ensure that the cultivated land area will not be reduced, select the permanent basic farmland protection area as the constraint, strictly control the quantity and direction of cultivated land transfer, restrict the conversion of basic farmland, and prevent it from being occupied in the process of rapid urbanization, so as to ensure the basic food security of the region. In the scenario of cultivated land protection, Qianjiang City takes food security as the main goal, and sets the cultivated land as non-convertible to other land types under the constraints of strong cultivated land protection policies. At the same time, considering the positive driving effect of cultivated land protection, most other land use types are set to be convertible into cultivated land.

Scenario 3 (Ecological protect scenario): in the context of ecological civilization construction and green development, with ecological benefit priority as the main development goal, strengthen the comprehensive improvement of natural resources, ensure ecological functions, protect environmental quality and safety, make rational use of natural resources, strengthen ecological protection, and reduce the negative effect of urban disorderly expansion on the external ecosystem. Select nature reserves, wetland parks, forest land and water areas as constraints, strictly protect ecological land, such as forest land, grassland and water areas, build ecological barriers, limit the use conversion of ecological control areas and ecologically sensitive areas, and improve the quality of regional ecological environment. Under the scenario of ecological protection, Qianjiang City takes ecological security as the main goal, focuses on the protection of ecological land and ensures that the ecosystem can provide sufficient services. Therefore, most other land use types are set to be converted into grassland or forest land only in one direction. At the same time, it is necessary to give due consideration to food security and economic development and set the land use types other than forest land, grassland and water area to be convertible into cultivated land and construction land.

#### 3.3.3. Land Use Simulation Parameter Setting

The land spatial demand document mainly includes the number of different land types (grid number) to simulate future years. Most existing research uses the transfer matrix area and selects the linear interpolation method to simulate and predict the demand of different land types in future years in proportion. This paper assumes that the demand of different land types in the same interval period increases steadily, adopts the land use transfer probability from 2005 to 2020, and takes 2020 as the base period, and uses a Markov model to obtain the land space demand target in the state of natural evolution in 2035 through the annual average transfer probability matrix.

Due to the process of urbanization and the continuous growth of population and GDP, the construction land in Qianjiang City shows a rapid increase trend under three scenarios (Table 8). By 2035, the natural development scenario has increased by 27.7 km^2^, the ecological protection scenario has increased by 21.66 km^2^, while the cultivated land protection scenario has increased by only 18.19 km^2^. The cultivated land area is constantly decreasing. By 2035, the cultivated land will be reduced by 44.60 km^2^ under the natural development scenario (ND), 44.03 km^2^ under the ecological protect scenario (EP), and the reduction under the cultivated land protection scenario is the least (30.3 km^2^). Under the three development scenarios, grassland increased slightly. Under the three scenarios, the woodland showed an increasing trend. Under the scenario of ecological protection, the forest area has increased significantly, with an increase of 6.16 km^2^ by 2035. Under the combined impact of climate change and socio-economic development, the water area has increased under the three scenarios. Affected by the development of other land use types, the unused land area has decreased to a certain extent.

The neighborhood weight parameter mainly reflects the occupation intensity of land use types under the influence of natural environment and human activities and reflects the expansion ability of different land units in the neighborhood. The neighborhood weight parameter is between 0 and 1 (Table 9). The number 1 indicates that it cannot be converted, 0 indicates that it is very easy to convert, and the closer its value is to 1, the more stable the land type is, and it is not easy to convert to other land. Conversely, the closer its value is to 0, the easier it is to convert. Qianjiang City is located in the core area of Jianghan Plain. The cultivated land resources in the area are of great significance to social and economic development and ecological environment protection, and it is generally difficult to convert. With reference to the existing neighborhood weight parameter setting results [43], the land use types are sorted and assigned based on the expansion capacity and corrected according to the actual situation of Qianjiang City.

#### 3.3.4. Simulation Results of Future Land Use Change

**Figure 9:**
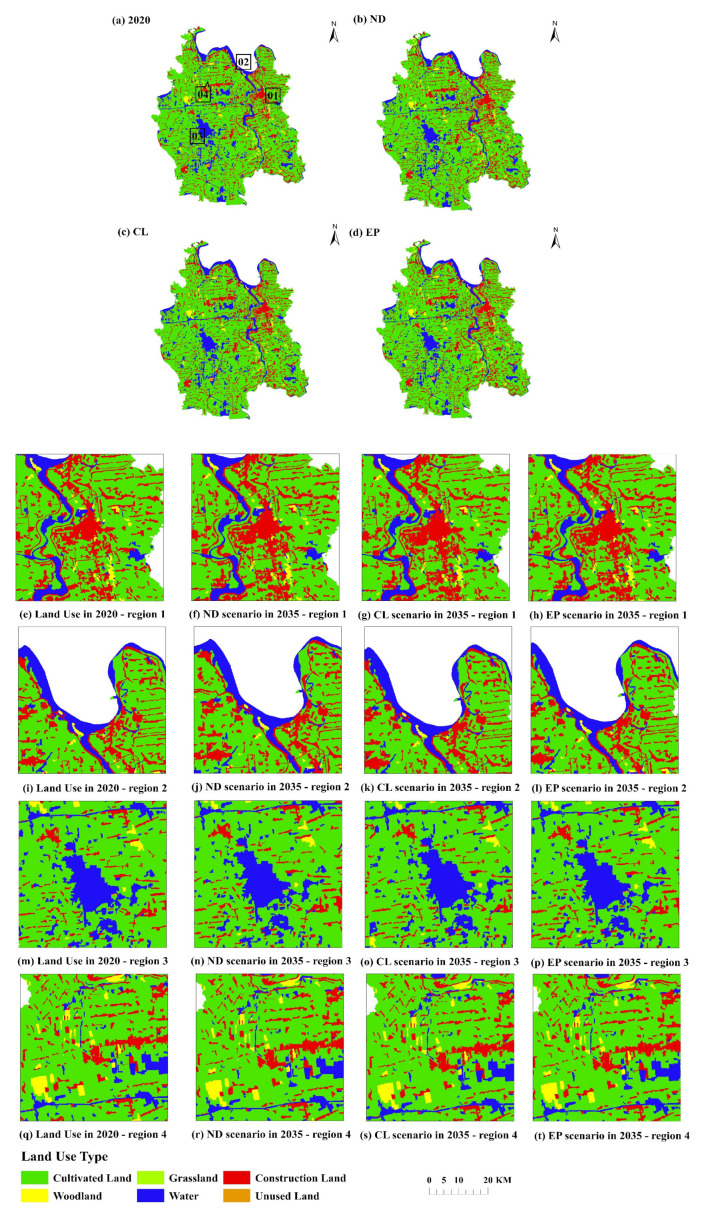
Land use in 2020 and simulation results of three different scenarios in 2035.

The prediction results of land use demand under three different development scenarios are respectively substituted into the flus model, and the actual land use data and driving factor data in 2020 are used as the initial data to simulate the future spatial distribution of land use in Qianjiang City. The simulation results of the final simulation year (2035) under three scenarios are compared, and four representative regions in the figure below are selected to display the results (Figure 9).

Area 1 shows the center of Qianjiang City, which is mainly used to show the distribution difference of construction land in Qianjiang City. Compared with the actual land use model in 2020, the CL scenario is mainly due to the restriction of basic farmland in other places, resulting in the expansion of the downtown area. The most important thing in the ND scenario is the occupation of the surrounding land by the expansion of construction land, but the expansion range of construction land is much smaller than that in the CL scenario. The EP scenario is mainly manifested in the transformation of grassland, cultivated land and even unused land around the original forest land to forest land, and the expansion of construction land slower than the CL scenario.

Area 2 shows an area near the Hanjiang river dominated by water and cultivated land, mixed with some construction land and a few forest patches. It is mainly used to show the mutual changes of cultivated land, grassland and forest land in the basin under different scenarios. Compared with the actual land use pattern in 2020, the most important land use change in the ND scenario is the erosion of construction land to cultivated land. The CL scenario is mainly the transformation from cultivated land to construction, but the change is larger than that in the BD scenario. In the HD scenario, due to the limitation of ecological protection areas, a considerable part of grassland and cultivated land are converted to construction land.

Area 3 shows Huiwan lake and its surrounding areas. In the economic development-oriented ND scenario, in the next ten years, it is mainly reflected in the expansion of construction land and the corresponding reduction of cultivated land. There is a similar performance in the CL scenario that continues the historical trend, and the change speed is relatively slow. In these two cases, the rapid expansion of construction land may be the demand of social and economic development and population growth for infrastructure construction. In contrast, in the EP scenario, due to the government’s attention to ecological and environmental protection and the pursuit of high-quality development, the urban expansion in this scenario is slower than that in the CL scenario.

Area 4 is located in the rural residential area in the north of Qianjiang City, which is mainly composed of cultivated land, forest land and construction land, showing the changes of forest tillage ecotone under different scenarios. Compared with the land use pattern in 2020, the changes of forest and cultivated land in the region under the ND scenario and the CL scenario are small, mainly due to a small amount of mutual transformation. In the EP development scenario, the most significant change is the occupation of cultivated land by the rapid expansion of water area.

#### 3.3.5. Conflict Simulation of Future “Production-Living-Ecological” Space

**Figure 10:**
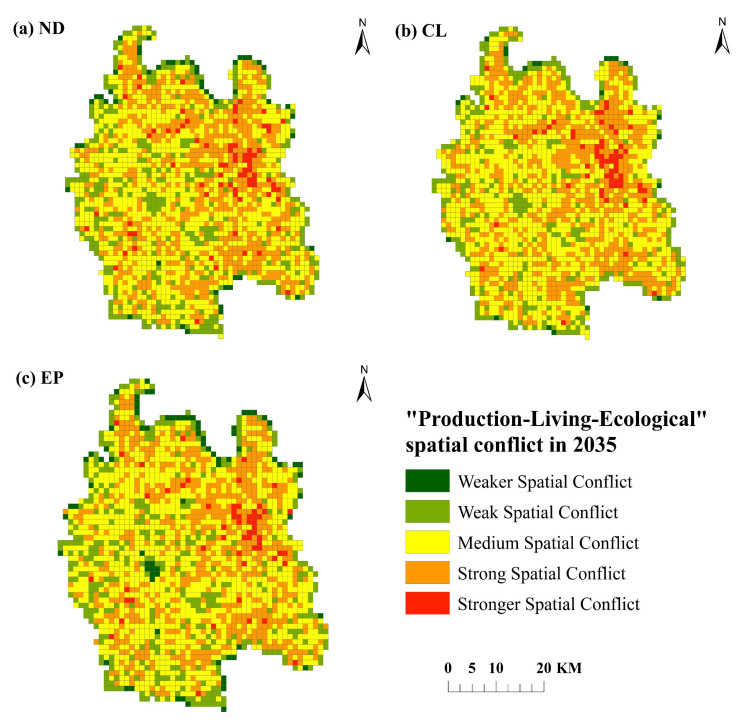
Spatial conflict results of “production-living-ecological” under three scenarios in 2035.

The spatial conflict measurement model of “production-living-ecological” is used to calculate the spatial conflict level of “production-living-ecological” in Qianjiang City under three scenarios (see Table 10 and Figure 10). The results show that in 2035, the spatial conflict in Qianjiang City will be strengthened under the three scenarios, mainly at the level of medium and above. In the natural development scenario, in addition to expanding in the city center, strong spatial conflicts are also scattered in the locations of various towns and townships in Qianjiang City, which have also been strengthened, so that the number of strong spatial conflicts is the largest in the three scenarios. Under the scenario of cultivated land protection, the number of weak spatial conflicts is the least, and the number of medium spatial conflicts is the most. It is mainly affected by the stability and vulnerability of spatial patches, and the level of spatial conflict is relatively high. In the ecological protection scenario, due to the limitation of ecological protection areas, the construction land cannot expand to these spaces, so that the number of weak space conflicts is the largest and the number of strong space conflicts is the least.

### 3.4. The Functional Zoning of “Production-Living-Ecological” Space

Because Qianjiang City is located in the hinterland of Jianghan Plain, there is a large amount of cultivated land. In the future development process, cultivated land protection is the most likely scenario. Therefore, based on the comparison of the current situation conflict of “production-living-ecological” space in 2020 and the degree of “production-living-ecological” space conflict of the cultivated land protection scenario in 2035, this paper analyzes the changing direction of “production-living-ecological” space conflict, so as to put forward optimization suggestions for the existing land use pattern. According to the analysis of “production-living-ecological” space conflict transfer matrix, the change can be obtained (as shown in Table 11). The box in Table 11 indicates that this category actually exists during the conversion process. For example, 11 represents the transition from the winner spatial conflict in 2020 to the winner spatial conflict in 2035, and 41 indicates the transition from strong spatial conflict in 2020 to threat spatial conflict in 2035.

**Figure 11:**
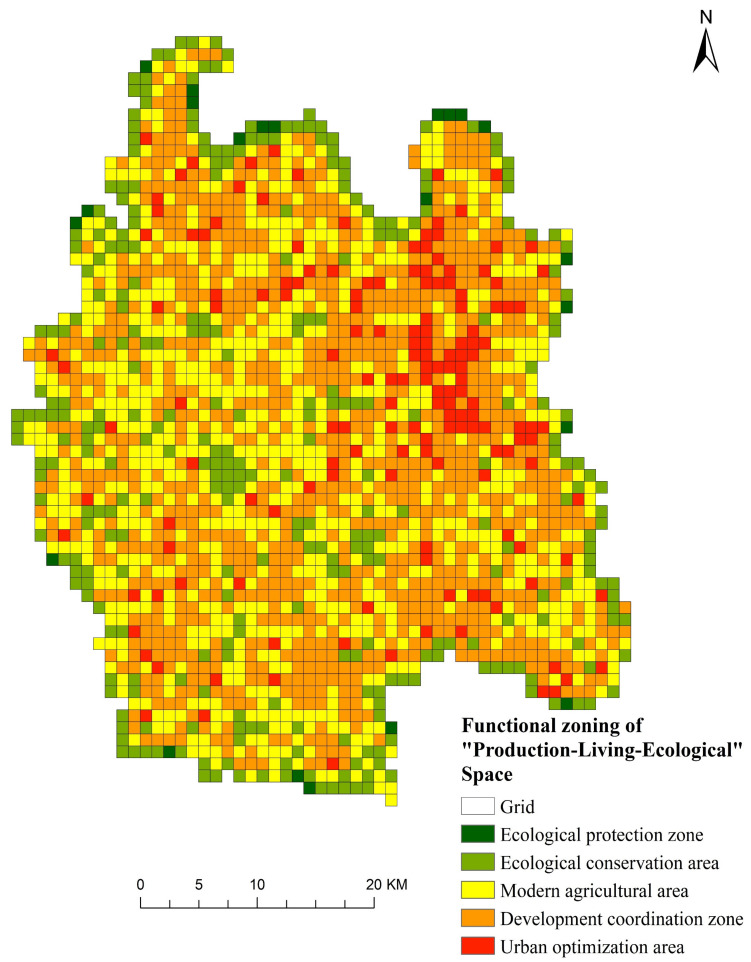
Functional zoning of “production-living-ecological” space.

According to the change degree of conflict transformation, 15 change types are divided into five functional zones, namely ecological protection zone, ecological conservation zone, modern agriculture zone, urban–rural development coordination zone and urban optimization zone (as shown in Table 12 and Figure 11). The conflict level in the ecological protection area is the lowest, and it is very important to maintain the ecological environment. It is vulnerable to damage and needs to be protected. The conflict level in the ecological conservation area is low, and includes water areas, forest lands and other land types that are of great significance to the maintenance of the ecological environment. The ecological protection and development model should be implemented. The level of conflict in the modern agricultural area is mainly medium conflict. A large number of cultivated lands, forest land and other land in this area are located in the periphery of construction land, and the conflict is controllable. Regional optimization can be carried out to alleviate the conflict. The urban–rural development coordination area is dominated by strong spatial conflict, which is located at the edge of the serious out of control level, and the area dominated by construction land is mostly the transition zone between urban and rural areas, which is easily affected. The urban optimization area is dominated by strong spatial conflict. The construction land in this area has expanded seriously in recent years and continuously eroded the surrounding land resources. Therefore, it is necessary to reasonably optimize the urban structure and improve the utilization rate of spatial resources.

## 4. Discussion

### 4.1. Deepening Understanding of Space Conflict

With the accelerating process of industrialization, the spatial form and structure of urbanization are undergoing drastic changes, resulting in the high-intensity development and utilization of spatial resources, further resulting in a series of “spatial conflicts”, such as the orderly expansion of urban construction land and the imbalance of spatial structure between agricultural land and ecological land [2]. Once the space conflict is out of control, it will lead to problems by affecting the diversity of space resources, destroying the stability of space structure, increasing the vulnerability of space resources and weakening the recovery of space resources, such as the mismatch and disorder of space development, the imbalance of ecosystem, the increase in environmental pollution, and the instability of social development [2].

At present, the systematic research on space conflict is still relatively limited in China. Only the geospatial research from the perspectives of geography, planning, ecology and economics indirectly reflects the understanding of spatial conflict [2]. From the perspective of geography and planning, some scholars have analyzed the existence of spatial conflict and its disciplinary significance from the aspects of regional deprivation [44], spatial competition [45], spatial integration [46] and spatial regulation [47]. Some scholars have used some classical theories in the field of ecology and economics, such as ecological space theory [48], niche situation theory [49] and regional spillover theory [50], for reference, and applied them to the research of regional space optimization, providing a reference for the research on the formation mechanism and regulation of spatial conflict scholars’ research on land use conflict is more in-depth and systematic, which can be divided into the following five aspects: first, the causes of land use conflict [51]. Special land system [52], scarce land resources [53] and multi-directional land use [54] are regarded to be the main sources of land use conflicts. The second is the types of land use conflicts [55]. Many scholars mainly classify land use conflicts from different research perspectives, including conflict areas [56,57], conflict processes [58,59], conflict consequences [60,61,62], etc. The third is the identification of land use conflict [63]. The basis and premise of preventing and solving land use conflict according to local conditions is to scientifically identify the potential occurrence area of land use conflict and diagnose its intensity.

Because the land use system is a giant “Nature-Society-Economy” complex system, and the land use conflict is the result of the comprehensive action of social economy, resources and environment, human activities and other factors, the application of different research methods can more scientifically identify the spatial process of land use pattern change in different regions and diagnose the impact of land use activities on nature, society and economy. Fourth, the evolution of land use conflict [64]. The change of institutional, social and economic environment makes the land use scenario evolve continuously and show a certain life cycle [65]. Fifth, reconciliation of land use conflicts [66]. Conflict reconciliation is the ownership of land use conflict research. The formulation of a land management plan needs to comprehensively use a variety of ways to coordinate the interest choice and goal orientation of relevant subjects [66]. In the future, China will further accelerate the process of urbanization. Without effective guidance, rapid urbanization will inevitably lead to the intensification of spatial conflict, directly affect the stability and sustainability of the regional “economy society ecology” composite system, and even threaten the regional economic security, social security and ecological security [2]. The manifestation, formation mechanism and influencing factors of space conflict are very complex, involving resources, environment, society, economy and other aspects. This paper has not studied the formation causes, classification forms, evolution characteristics and impact benefits of space conflict. Therefore, the next research work needs to deeply explore the causes, regulation mechanism and mode of space conflict.

### 4.2. Deficiencies and Future Direction

In order to simulate land use change, many scholars have developed many models, such as the CLUE-S model [39,67,68], FLUS model [42,43,69] and PLUS model [70,71]. Recent studies have shown that most spatial models still have a high degree of uncertainty, which indicates no single model or method can fully describe the different processes of land use on all spatiotemporal scales [72,73,74]. In this study, a Markov model can describe the direction of land use change by considering the impact of relevant factors on land use demand, predict the future land demand of land use types, and allocate the predicted land demand to geospatial through FLUS model, so as to realize land use spatial prediction. The results of this research show that the combination of a Markov model and FLUS model can predict the possible land use change under different scenarios, so as to provide scientific basis and decision support for land management and planning.

The complexity of the LULC system requires that the selection of driving factors of the FLUS model needs to be based on the theoretical relationship between driving factors and land use [42]. We select the relevant driving factors affecting land use change from three aspects: socio-economic factors, natural environmental factors and accessibility factors, but there are some other factors that have not been selected to avoid over fitting of the model. The selection of variables and indicators may lead to the difference of simulation results or model parameters to a certain extent, which may have a certain impact on the driving factors and prediction of LULC change [63]. The FLUS model can reflect the complexity of future land use change, but different situations may occur if different land prediction models are selected. In many cases, it is appropriate to use different land use simulation models, such as grey correlation degree [75], system dynamics model [76] or multi-agent system model [77], to study the same area, and then compare the accuracy of the prediction results with the actual situation.

However, in this research, we did not consider the impact of various policy factors in the process of land use change simulation. In this study, the terrain of Qianjiang City is relatively flat, which only represents one type of city. If the method and model of this study are extended to other cities, the impact factors should be selected according to the natural, social and economic conditions of the studied cities, so as to make the research results more reliable. In further research, multiple models can be used for combined prediction and comparison, and the combination of regional spatial factors, land adaptability factors, socio-economic and policy related factors can be considered to make the simulation results more accurate, so as to provide advice for the government’s land use decisions.

### 4.3. Policy Enlightenment

The level of spatial conflict in Qianjiang City shows an upward trend, the proportion of medium and above conflict units gradually increases, and the conflict level in the study area is gradually dominated by strong spatial conflict. On the one hand, rapid urbanization has promoted social and economic development, but on the other hand, it has also brought a series of environmental problems, such as the decline of cultivated land area and quality, the loss of biological habitat and the destruction of ecosystem. Therefore, the formulation of reasonable land use policies is very important for the improvement of human well-being and the protection of ecological environment in the region.

According to the results of land use simulation and spatial conflict model under multiple scenarios, three aspects of land use need to be considered in the future development of the city: (1) The gradual acceleration of urbanization will inevitably lead to the rapid growth of construction land in the city. Therefore, it is necessary to put the intensive development of land into the key consideration in the urban optimization area. (2) The acceleration of urbanization and the expansion of built-up areas lead to the occupation of a large number of high-quality cultivated land in development coordination areas, which will bring great pressure to maintain food production and increase the area of cultivated land. Therefore, we must strengthen the protection of basic farmland, improve food production conditions and increase grain yield per unit area to ensure food security. (3) Ecological protection zones and ecological conservation areas need to be protected. In Qianjiang City, vegetation and water are natural ecological barriers to ensure ecological security in this area. Therefore, targeted policies are needed to ensure ecological space, such as national nature reserve management regulations, afforestation and other measures. 

## 5. Conclusions

This study studies the spatiotemporal characteristics of the spatial conflict in Qianjiang City from 2000 to 2020 and uses the FLUS model to predict the spatial conflict in 2035 under multiple scenarios, and finally obtains the functional zoning in Qianjiang City. The main conclusions of this study are summarized as follows:

(1) According to land use data, from 2000 to 2020, the cultivated land and grassland area in Qianjiang City showed the characteristics of different scales and continuous reduction. The change range of cultivated land, forest land, grassland and water area are also large, with the change rates of 4.11%, 25.50% and 11.39%, respectively.

(2) The proportion of “production-ecological” space is the highest from 2000 to 2020 in terms of quantity structure, exceeding 74% of the total land area of Qianjiang City. The second is “living-production” space and “ecological-production” space, with their total area accounting for 22–24%.

(3) Overall, from 2000 to 2020, the spatial conflict in Qianjiang City is mainly at the level of medium and below, and the spatial conflict unit accounts for 54.91%. The spatial conflict level in Qianjiang City showed an upward trend, the unit proportion of medium and above conflicts gradually increased, and the conflict level in the research area gradually became dominated by strong conflicts.

(4) In this study, the FLUS model has good simulation effect and meets the requirements of rapid urbanization, national policy of returning farmland to forest and grassland and water resources protection. 

The results show that in 2035, the spatial conflict in Qianjiang City will be strengthened under the three scenarios, mainly at the level of medium and above. Therefore, it is very important to formulate reasonable land use policies for the protection of ecological land and ecological security. The government should pay attention to intensive land development, protect basic farmland and protect ecological land. However, this study does not consider the impact of individual behavior, individual preferences and government policies in land use change. Therefore, in the future research, the simulation and quantification of government policies can be added to the model so as to provide a scientific support for reasonable decision-making.

## Figures and Tables

**Table 1:** The division framework of “production-living-ecological” space.

Class 1	Class 2
ecological space	River, lake, barren grassland, coastal beach, sandy land and bare land
“ecological-production” space	Woodland, shrub woodland, other woodland, land for natural scenery, and water surface of reservoirs and ponds
“production-ecological” space	Paddy field, dry land, irrigated land, orchard and other gardens
“living-production” space	Towns, villages, mining land, transportation land, hydraulic construction land, ports and wharfs, special land

**Table 2:** Data source and description.

Data Type		Name	Source
Basic data	Administrative boundary	Administrative Region	Resource and Environment Science and Data Center (https://www.resdc.cn, accessed on 1 May 2020)
	Land use	Land use data	
Spatial driving factor	Socio economic drivers	Population density	WorldPoP database (https://www.worldpop.org/methods/populations, accessed on 1 May 2020)
		Night light data	VIIRS nighttime lights (https://eogdata.mines.edu/product/vnl/, accessed on 1 May 2020)
		Grid GDP	Grid data set of spatial distribution of China’s GDP (https://www.resdc.cn, accessed on 1 May 2020)
	Natural environment drivers	Elevation	Geospatial data cloud (https://www.gscloud.cn, accessed on 1 May 2020)
		Slope	
		Slope aspect	
		Annual average temperature	National Meteorological Science Data Center (http://data.cma.cn, accessed on 1 May 2020)
		Annual average precipitation	
	Accessibility drivers	Distance from main road	The vector data come from the land survey database of Qianjiang City, and the relevant results are calculated by European distance
		Distance from railway	
		Distance from river	
		Distance from residential area	
		Distance from city	
Spatial restriction factor	Ecological Reserve		Through land use data extraction
	Basic farmland area		Qianjiang City Land Use Planning Database (2006–2020)

**Table 3:** The change characteristics of land use structure in different periods (unit: km^2^).

Year	Cultivated Land	Woodland	Grassland	Water	Construction Land	Unused Land
2000	1561.89	25.48	0.06	192.14	234.60	2.83
2005	1549.37	25.22	0.07	201.56	238.29	2.50
2010	1508.80	35.04	0.00	212.02	261.04	0.14
2015	1497.35	34.45	0.00	211.83	273.19	0.14
2020	1497.68	31.97	0.11	214.04	269.93	2.36
2000–2005	−12.52	−0.26	0.00	9.42	3.69	−0.33
	−0.80%	−1.01%	5.63%	4.90%	1.57%	−11.68%
2005–2010	−40.57	9.82	−0.07	10.46	22.75	−2.36
	−2.62%	38.95%	−100.00%	5.19%	9.55%	−94.31%
2010–2015	−11.45	−0.59	0.00	−0.19	12.16	0.00
	−0.76%	−1.70%	0.00%	−0.09%	4.66%	0.00%
2015–2020	0.33	−2.48	0.11	2.20	−3.27	2.22
	0.02%	−7.19%	0.00%	1.04%	−1.20%	1558.86%
2000–2020	−64.21	6.50	0.05	21.89	35.32	−0.47
	−4.11%	25.50%	78.87%	11.39%	15.06%	−16.56%

**Table 4:** The change characteristics of “production-living-ecological” space in different periods (unit: km^2^).

Year	“Living-Production” Space	“Production-Ecological” Space	“Ecological-Production” Space	Ecological Space
2000	234.60	1561.95	217.62	2.83
2005	238.29	1549.44	226.78	2.50
2010	261.04	1508.80	247.06	0.14
2015	273.19	1497.35	246.28	0.14
2020	269.93	1497.79	246.01	2.36
2000–2005	3.69	−12.52	9.16	−0.33
	1.57%	−0.80%	4.21%	−11.65%
2005–2010	22.75	−40.64	20.28	−2.36
	9.55%	−2.62%	8.94%	−94.31%
2010–2015	12.16	−11.45	−0.78	0.00
	4.66%	−0.76%	−0.32%	0.00%
2015–2020	−3.27	0.44	−0.27	2.22
	−1.20%	0.03%	−0.11%	1558.86%
2000–2020	35.32	−64.16	28.39	−0.47
	15.06%	−4.11%	13.05%	−16.56%

**Table 5:** Comprehensive index table of “production-living-ecological” spatial conflict in Qianjiang City.

Conflict Type	Conflict Classification	Number and Proportion of Conflict Space Units				
		2000	2005	2010	2015	2020
Weaker Spatial Conflict	0–0.2	55	16	12	15	14
		2.51%	0.73%	0.55%	0.68%	0.64%
Weak Spatial Conflict	0.2–0.4	449	217	241	238	202
		20.47%	9.90%	10.99%	10.85%	9.21%
Medium Spatial Conflict	0.4–0.6	893	1009	951	919	897
		40.72%	46.01%	43.37%	41.91%	40.90%
Strong Spatial Conflict	0.6–0.8	737	877	914	926	935
		33.61%	39.99%	41.68%	42.23%	42.64%
Stronger Spatial Conflict	0.8–1.0	59	74	75	95	145
		2.69%	3.37%	3.42%	4.33%	6.61%
Total		2193	2193	2193	2193	2193

**Table 6:** Distribution of spatial conflicts at different levels on different land types.

Year	Conflict Type	Weaker Spatial Conflict	Weak Spatial Conflict	Medium Spatial Conflict	Strong Spatial Conflict	Stronger Spatial Conflict
2000	“Living-Production” space	0.01%	0.21%	3.77%	6.74%	0.90%
	“Production-Ecological” space	0.21%	15.76%	33.71%	25.92%	1.84%
	“Ecological-Production” space	0.12%	2.02%	4.74%	3.71%	0.20%
	Ecological space	0.00%	0.07%	0.07%	0.00%	0.00%
2005	“Living-Production” space	0.00%	0.06%	2.78%	7.79%	1.19%
	“Production-Ecological” space	0.02%	3.80%	39.15%	31.55%	2.31%
	“Ecological-Production” space	0.06%	2.06%	5.25%	3.68%	0.19%
	Ecological space	0.00%	0.06%	0.06%	0.00%	0.00%
2010	“Living-Production” space	0.00%	0.07%	3.03%	8.62%	1.22%
	“Production-Ecological” space	0.02%	4.31%	35.91%	32.26%	2.31%
	“Ecological-Production” space	0.06%	2.75%	5.12%	4.12%	0.20%
	Ecological space	0.00%	0.00%	0.00%	0.00%	0.00%
2015	“Living-Production” space	0.00%	0.07%	2.90%	8.99%	1.59%
	“Production-Ecological” space	0.02%	4.26%	34.56%	32.44%	2.96%
	“Ecological-Production” space	0.06%	2.66%	5.14%	4.17%	0.19%
	Ecological space	0.00%	0.00%	0.00%	0.00%	0.00%
2020	“Living-Production” space	0.00%	0.05%	2.33%	8.51%	2.49%
	“Production-Ecological” space	0.01%	3.48%	33.69%	32.70%	4.40%
	“Ecological-Production” space	0.06%	2.17%	5.27%	4.40%	0.30%
	Ecological space	0.00%	0.06%	0.05%	0.01%	0.00%

**Table 7:** Cost matrix setting in multi scenario simulation.

	Natural Development Scenario (ND)						Cultivated Land Protection Scenario (CL)						Ecological Protect Scenario (EP)					
	A ^1^	B	C	D	E	F	A	B	C	D	E	F	A	B	C	D	E	F
A	1	1	1	0	1	0	1	1	1	0	1	1	1	1	1	0	1	0
B	1	1	1	0	1	1	1	1	1	0	1	1	0	1	0	0	0	0
C	1	1	1	0	1	1	1	1	1	0	1	1	0	0	1	0	0	0
D	0	0	0	1	1	1	0	0	0	1	0	1	0	0	0	1	0	1
E	0	0	0	0	1	0	0	0	0	0	1	0	0	1	1	0	1	0
F	1	1	1	0	1	1	1	1	1	0	1	1	1	1	1	0	1	1

^1^ A, B, C, D, E and F, respectively, represent cultivated land, forest land, grassland, water, construction land, and unused land. The number 1 indicates that conversion is allowed. The number 0 indicates that conversion is not allowed.

**Table 8:** Demand forecast of land spatial layout in Qianjiang City in 2035.

Year	Cultivated Land	Woodland	Grassland	Water	Construction Land	Unused Land
Actual land use in 2010	1508.80	35.04	0.00	212.02	261.04	0.14
Actual land use in 2015	1497.35	34.45	0.00	211.83	273.19	0.14
Actual land use in 2020	1497.68	31.97	0.11	214.04	269.93	2.36
Natural development scenario (ND)	1453.65	37.67	0.15	225.29	297.63	2.24
Ecological protect scenario (EP)	1458.42	38.13	0.15	226.10	291.59	2.24
Cultivated land protection scenario (CL)	1467.38	33.24	0.15	225.51	288.12	2.24

**Table 9:** Neighborhood weight parameter table.

Land Use Type	Cultivated Land	Woodland	Grassland	Water	Construction Land	Unused Land
Neighborhood weight	0.56	0.25	0.36	0.43	1	0.25

**Table 10:** Number and proportion of spatial conflict units of “production-living-ecological” under multi situation analysis.

Conflict Type	Conflict Classification	Multi Scenario Analysis		
		Natural Development Scenario	Cultivated Land Protection Scenario	Ecological Protection Scenario
Weaker Spatial Conflict	0–0.2	52	22	65
		2.37%	1.00%	2.96%
Weak Spatial Conflict	0.2–0.4	383	322	383
		17.46%	14.68%	17.46%
Medium Spatial Conflict	0.4–0.6	962	1043	1036
		43.87%	47.56%	47.24%
Strong Spatial Conflict	0.6–0.8	716	740	649
		32.65%	33.74%	29.59%
Stronger Spatial Conflict	0.8–1.0	80	66	60
		3.65%	3.01%	2.74%
Total		2193	2193	2193

**Table 11:** The “production-living-ecological” conflict function zoning rules.

Conflict Level			Year 2035				
			Weaker Spatial Conflict (1)	Weak Spatial Conflict (2)	Medium Spatial Conflict (3)	Strong Spatial Conflict (4)	Stronger Spatial Conflict (5)
			Weak Conflict	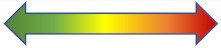			Strong Conflict
Year 2020	Weaker Spatial Conflict (1)	**Weak** **Conflict**	11	12 ^1^	13	14	15
	Weak Spatial Conflict (2)	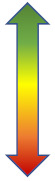	21	22	23	24	25
	Medium Spatial Conflict (3)		31	32	33	34	35
	Strong Spatial Conflict (4)		41	42	43	44	45
	Stronger Spatial Conflict (5)	**Strong** **Conflict**	51	52	53	54	55

^1^: 1 stands for weaker spatial conflict, 2 stands for weak spatial conflict, 3 stands for medium spatial conflict, 4 stands for strong spatial conflict, and 5 stands for stronger spatial conflict.

**Table 12:** “production-living-ecological” space conflict functional area.

Functional Area	Category	Main Problems	Measures
Ecological protection zone	11, 21, 41	Important ecological protection areas need to be protected	Establish ecological protection areas
Ecological conservation zone	22, 32, 42	It has the function of regulating climate and maintaining ecosystem stability	Adopt an ecological protection development model
Modern agricultural zone	13, 23, 33	The relationship between man and land is complex and the ecological environment is easily damaged	Protect cultivated land and develop efficient agriculture
Development coordination zone	43, 34, 44	The advantages of urban land use are obvious, and a large number of surrounding land resources are eroded	Tap urban space resources
Urban optimization zone	45, 54, 55	The utilization rate of land resources is poor, and most of the land idle	Optimize urban layout and improve land use efficiency

## Data Availability

Not applicable.
